# Investigating Transcriptomic Induction of Resistance and/or Virulence in *Listeria monocytogenes* Cells Surviving Sublethal Antimicrobial Exposure

**DOI:** 10.3390/foods10102382

**Published:** 2021-10-08

**Authors:** Eleni-Anna Kokkoni, Nikolaos Andritsos, Christina Sakarikou, Sofia Michailidou, Anagnostis Argiriou, Efstathios Giaouris

**Affiliations:** 1 Department of Food Science and Nutrition, School of the Environment, University of the Aegean, Ierou Lochou 10 & Makrygianni, 81400 Myrina, Greece; fns15037@fns.aegean.gr (E.-A.K.); nickandritsos@aegean.gr (N.A.); chsakarikou@aegean.gr (C.S.); sofia_micha28@certh.gr (S.M.); argyriou@aegean.gr (A.A.); 2 Athens Analysis Laboratories S.A., Microbiology Laboratory, Nafpliou 29, 14452 Metamorfosi, Greece; 3 Centre for Research and Technology Hellas (CERTH), Institute of Applied Biosciences, 57001 Thessaloniki, Greece

**Keywords:** *Listeria monocytogenes*, benzalkonium chloride, thymol, ampicillin, sublethal antimicrobial exposure, survival, gene expression, stress response, virulence

## Abstract

The potential transcriptomic induction of resistance and/or virulence in two *L. monocytogenes* strains belonging to the most frequent listeriosis-associated serovars (i.e., 1/2a and 4b), following their sublethal antimicrobial exposure, was studied through qPCR determination of the relative expression of 10 selected related genes (i.e., *groEL*, *hly*, *iap*, *inlA*, *inlB*, *lisK*, *mdrD*, *mdrL*, *prfA*, and *sigB*). To induce sublethal stress, three common antimicrobials (i.e., benzalkonium chloride, thymol, and ampicillin) were individually applied for 2 h at 37 °C against stationary phase cells of each strain, each at a sublethal concentration. In general, the expression of most of the studied genes remained either stable or was significantly downregulated following the antimicrobial exposure, with some strain-specific differences to be yet recorded. Thymol provoked downregulation of most of the studied genes, significantly limiting the expression of 6/10 and 4/10 genes in the strains of ser. 1/2a and ser. 4b, respectively, including those coding for the master regulators of stress response and virulence (SigB and PrfA, respectively), in both strains. At the same time, the two genes coding for the invasion internalin proteins (*InlA* and *InlB*), with crucial role in the onset of *L. monocytogenes* pathogenesis, were both importantly upregulated in ser. 4b strain. The results obtained increase our knowledge of the stress physiology of *L. monocytogenes* under certain sublethal antimicrobial conditions that could be encountered within the food chain and in clinical settings, and may assist in better and more effective mitigation strategies.

## 1. Introduction

*Listeria monocytogenes* is an important Gram-positive pathogenic bacterium provoking listeriosis, a rare but quite life-threatening foodborne disease mainly for those belonging to vulnerable groups, such as the elderly and immunocompromised [1]. Based on the latest available data for Europe, 2621 confirmed cases of human listeriosis were recorded in 2019, resulting in 1234 hospitalizations and eventually 300 deaths, presenting an enormous case fatality ratio of 17.6% [2]. In the United States, *L. monocytogenes* is estimated to cause approximately 1600 cases of foodborne illness annually, resulting in 1500 hospitalizations (i.e., 94% hospitalization rate) and more than 250 deaths, with a similar death rate to that recorded in Europe, which for the susceptible individuals is further increased to 25–30% [3]. *L. monocytogenes* is known as a highly versatile microorganism that can skillfully adjust its physiology to confront various stress conditions, including high acidity or alkalinity, high osmotic concentration, existence of reactive oxygen species (ROS), increased or low temperature, allowing this way its survival and persistence in a wide range of environmental, food-associated, and clinical conditions [4]. That remarkable adaptation to stress is accomplished through global changes in many cellular constituents, including modifications in gene expression and protein activities [5]. All those changes enable this soil-living bacterium to successfully switch from a harmless saphrophyte to a powerful intracellular pathogen [6].

Many of the survival mechanisms that are exploited by *L. monocytogenes* are known to be controlled by the stress-inducible alternative sigma factor B (*σ*^B^), which is the master regulator of the general stress response (GSR) in that pathogen [7]. It is thus known that *σ*^B^ controls in *L. monocytogenes* the expression of more than 300 genes, while it seems that it plays the same important role in several other Gram-positive foodborne pathogens, such as *Bacillus cereus* and *Staphylococcus aureus* [8]. Following consumption of the contaminated food and the survival of *L. monocytogenes* under the hostile conditions of the gastrointestinal (GI) tract [9,10], the subsequent victorious transit of the bacterium through the intestinal epithelial barrier, its intracellular growth, further proliferation, and dissemination relies on multiple virulence factors, the expression of the majority of which is under the control of the master regulator of virulence PrfA [11,12]. Alarmingly, *L. monocytogenes* can not only survive long-term in a stationary phase outside the host without compromising its virulence [13], but at the same time a complex overlap and crosstalk between *σ*^Β^ and PrfA regulons also exist at transcriptional, post-transcriptional, and protein activity levels. In this way bacterium succeeds achieving a peculiar balance and coordination between stress resistance and virulence skills, depending on the environment [14,15].

Up to now, many studies have selectively examined the expression of key stress response and/or virulence genes in *L. monocytogenes* cells that have either grown in foods such as fruits and vegetables [16,17], cheeses [18], raw and processed meats [19,20,21,22], and fish [23], or have been exposed to low temperatures, acid and/or salinity stresses [24,25,26,27,28], or even in a simulated gastrointestinal environment [29,30]. Undoubtedly, all these studies have provided valuable information on the physiology and pathogenesis of that bacterium under some critical food-associated circumstances, revealing in some cases a worrying increase in pathogenicity following such habituation [31]. It is also recognized that after repeated exposure to some antimicrobials, *L. monocytogenes* can adapt to them, and apart from surviving, these bacteria can also display cross-resistance to other antimicrobials and stresses other than those already adapted [32,33].

Indeed, sublethal antimicrobial concentrations could also be accidentally encountered following an ineffective sanitization program (e.g., due to the dilution of disinfectants in the environment, biodegradation, cellular entrapment in places that are not easily reached by the disinfectants, and biofilm formation) [34] or even applied on purpose. This last is the case for several chemical preservatives added to foods in low doses just to delay bacterial growth [35]. Riskily, sublethal concentrations of ampicillin have also been described to exist in the central nervous system (CNS), even following daily intravenous administration at high quantities (12 g), explaining the clinical failure of that antibiotic to treat this severe invasive case of listeriosis infection [36]. The stress-hardening that may appear in *L. monocytogenes* following such sublethal exposures should also contribute to the environmental persistence and spreading of that pathogen throughout the food chain [37]. However, only a few studies have investigated whether and in which way low concentrations of antimicrobial compounds can affect the physiology of that bacterium at the level of gene expression [38,39,40].

Considering all the above, the objective of the current study was to quantify the relative expression of some key stress response and/or virulence associated genes in two *L. monocytogenes* strains belonging to the most frequent listeriosis-associated serovars (i.e., 1/2a and 4b) [41], which survived after exposure to three common antimicrobials, belonging to different classes and which among others are used within the food industry and/or in clinical settings. These consisted of a general-purpose synthetic biocide (i.e., benzalkonium chloride; BAC), a natural terpenoid of plant origin (i.e., thymol; THY), and a broad-spectrum beta-lactam antibiotic (i.e., ampicillin; AMP). More specifically, BAC belongs to the family of quaternary ammonium compounds (QACs), which are membrane-active agents and among the most used disinfectants in industrial, healthcare, home, and cosmetics settings [42]. THY is found in rich quantities in the essential oils of thyme and oregano, as well as of several other related herbs, most native in the Mediterranean region, and this is well-known for its many biological and therapeutic properties, including broad-spectrum antimicrobial action [43]. Lastly, AMP is widely used to treat many bacterial infections, caused by either Gram-positive or -negative bacteria, inhibiting bacterial cell wall (peptidoglycan) biosynthesis [44]. In addition, this is currently included among the drugs of choice for the treatment of invasive listeriosis [45].

## 2. Materials and Methods

### 2.1. Bacterial Strains and Growth Conditions

The two tested *L. monocytogenes* strains were the foodborne AAL20066 (ser. 1/2a) and AAL20074 (ser. 4b) isolates deposited in the microbial culture collection of the Microbiology Laboratory in Athens Analysis Laboratories S.A. (AAL). Both strains were previously recovered from mixed fresh salads and were kept frozen long-term (at −80 °C) in Trypticase Soya Broth (TSB; Condalab, Torrejón de Ardoz, Madrid, Spain) containing 15% (*v/v*) glycerol. When needed for the experiments, each strain was streaked on to the surface of Tryptone Soya Agar (TSA; Oxoid, Thermo Fisher Specialty Diagnostics Ltd., Hampshire, UK) and incubated at 37 °C for 24 h (preculture). Working cultures were prepared by inoculating a colony from each preculture into 10 mL of fresh TSB and further incubating at 37 °C for 18 h. Bacteria from each of those final working cultures were collected by centrifugation (2000× *g* for 10 min at 4 °C), washed once with quarter-strength Ringer’s solution (Lab M, Heywood, Lancashire, UK), and finally suspended in 5 mL of the same solution (ca. 10^9^ CFU/mL). The purity of each cellular working suspension was verified through streaking on TSA plates.

### 2.2. Chemical Antimicrobials (BAC, THY and AMP)

BAC was bought from Acros Organics (Thermo Fisher Scientific, Geel, Belgium) (liquid, alkyl distribution from C_8_H_17_ to C_16_H_33_), THY was purchased from Penta Chemicals (Radiová, Prague, Czech Republic) (powder min. 99.0%, molar mass: 150.22 g/mol), while AMP was acquired from Cayman Chemicals (Ann Arbor, MI, USA) (crystalline solid ≥ 95% purity, molar mass: 371.4 g/mol). The stock solution of BAC (1% *v/v*) was prepared in sterile distilled water (dH_2_O), while those of THY and AMP (10% and 1% *w/v*, respectively) were prepared in absolute ethanol and were both subsequently filtrated by passing through disposable syringe filters (0.45 μm diameter; Whatman, Buckinghamshire, UK). All stock solutions were aliquoted and stored at −20 °C until needed for the experiments.

### 2.3. Determination of Minimum Inhibitory Concentration (MIC)

The MIC of AMP against the planktonic growth of each of the two bacterial strains was determined through the classical broth microdilution method, using sterile 96-well polystyrene flat-bottomed microtiter plates, as previously described [46]. In addition, the MICs of both BAC and THY had also been determined in that previous study. In sum, bacterial cultures of each strain (ca. 10^5^ CFU/mL) in TSB, containing 10 different increasing concentrations of the antibiotic (ranging from 0.063 to 5 μg/mL), were statically incubated at 37 °C for 24 h and were then checked for turbidity (as a visible indication of bacterial growth). Wells containing inoculated medium with the bacteria without the antibiotic and wells containing only sterile medium were used as positive and negative growth controls, respectively. For each concentration, two replicate wells were used, while the experiment was thrice repeated starting from independent bacterial cultures.

### 2.4. Sublethal Antimicrobial Exposure and RNA Extraction

For each tested strain and antimicrobial, the freshly saline cellular suspension (prepared as described in Section 2.1) was aliquoted in two Eppendorf^®^ tubes (2 mL in each one) and centrifuged (5000× *g* for 10 min at 4 °C). One of the two bacterial pellets was then suspended in 1 mL of the appropriate antimicrobial solution (i.e., 4.0 μg/mL BAC, 312.5 μg/mL THY, or 0.5 μg/mL AMP), while the second pellet was suspended in 1 mL of dH_2_O to be used as the untreated control sample. In the case of THY and AMP testing, the dH_2_O of the control sample also contained absolute ethanol at the concentration that existed in each working solution prepared for those two antimicrobials (i.e., 2812.5 and 50 μg/mL, for THY and AMP, respectively). Both samples (i.e., with the antimicrobial and its respective control) were incubated in a heating dry block for 2 h at 37 °C and were then immediately centrifuged (5000× *g* for 10 min at 4 °C). Supernatants were discarded and each pellet was washed with dH_2_O through an additional centrifugation step (5000× *g* for 10 min at 4 °C) to remove any antimicrobial residues. It should be noted that this washing procedure was sufficient for the efficient neutralization of each disinfectant, as this had been confirmed in preliminary experiments (through agar plating). Washed pellets were then placed on ice and directly used for RNA extraction using the RiboPure^TM^ -Bacteria Kit (Part Number: AM1925, Ambion, Life Technologies, Carlsbad, CA, USA). Eluted RNAs were treated with DNase I to remove any trace amounts of genomic DNA (gDNA), following the protocol guidelines, before measuring their absorbances at 260 and 280 nm to determine their concentrations and purities. One microgram of each extracted RNA sample was also run on electrophoresis (1.5% *w/v* TBE agarose gel; 100 V for 30 min) to verify its integrity, using the ssRNA Ladder (N0362S, 500–9000 bp, New England BioLabs Inc., Ipswich, MA, USA) as the molecular weight marker. The rest of each RNA sample was stored at −80 °C until its use as substrate for the subsequent reverse transcription (cDNA synthesis) reactions. Each antimicrobial exposure experiment was thrice repeated, starting each time from an independent bacterial culture and always using freshly prepared working antimicrobial solutions.

### 2.5. Reverse Transcription (cDNA Synthesis)

A cDNA synthesis was conducted starting from 500 ng of each RNA sample using the PrimeScript™ RT reagent Kit (Cat. #RR037A, Takara Bio Inc., Shiga, Japan). Both oligo dT and random hexamer primers were included in the reaction mixture (10 μL) at final concentrations of 25 and 50 pmol, respectively, according to the manufacturer’s instructions. For each RNA sample, a no-reverse transcription control (NRTC), which did not contain the reverse transcriptase enzyme (PrimeScript RT Enzyme Mix I), was also prepared to evaluate (i.e., in the later qPCR reactions) the presence of any residual gDNA. All RT reactions were performed in a PeqStar 96 HPL Gradient Thermocycler (Peqlab, VWR International GmbH, Darmstadt, Germany) by initially incubating at 37 °C for 15 min (for the RT reaction) and subsequently at 85 °C for 5 s (to inactivate reverse transcriptase). All resulting cDNAs were stored at −20 °C until their use as substrates in the subsequent qPCR analyses.

### 2.6. qPCR for Quantitation of mRNA Transcripts

Each cDNA template was used to quantify the expression of each gene of interest (including the ten targets and two additional reference genes; Table 1), for each bacterial strain and antimicrobial treatment and in relation to the respective untreated control, in qPCR reactions prepared using the PowerUp^TM^ SYBR^TM^ Green Universal 2X Master Mix (Cat. No. A25780, Applied Biosystems, Thermo Fisher Scientific, Waltham, MA, USA). Each reaction mixture contained 10 μL of PowerUp^TM^ SYBR^TM^ Green 2X Master Mix, 400 nM of each primer, 10 ng of cDNA template and PCR-grade water to a total volume of 20 μL. A no-template control (NTC) was always included in each assay to exclude any external DNA contamination. Real-time PCR was conducted on a QuantStudio™ 5 Real-Time PCR Instrument (Applied Biosystems). The PCR program consisted of two initial 2-min incubations, first at 50 °C for the uracil-DNA glycosylase (UDG) activation and the second at 95 °C for the activation of the (hot-start) Dual-Lock™ DNA polymerase, followed by 40 cycles of denaturation at 95 °C for 1 s and primer annealing/extension at 60 °C for 30 s (fast cycling mode). At the end of the amplification protocol, a melting curve analysis was also performed to confirm the specificity of each qPCR reaction (excluding any nonspecific amplification). This consisted of an initial step at 95 °C for 15 s (1.6 °C /s), a second step at 60 °C for 1 min (1.6 °C /s), and a final step at 95 °C for 15 s (0.15 °C /s). The threshold cycle (*C_T_*) for each reaction was calculated using the QuantStudio™ Design and Analysis Software v1.5.1 (Applied Biosystems). For each strain and antimicrobial treatment, the relative quantification of the expression of each target gene was finally performed using the classical comparative *ΔΔC_T_* method [47] in relation to the untreated control samples (i.e., with no antimicrobial exposure). Two reference (internal control) genes (i.e., *tuf*, *gap*) were always included in each assay, and were both used in parallel for the normalization of the qPCR data for any differences in the amount of total cDNA added to each reaction [48]. Both had been found to present the most consistent expression at both strains (exposed at the different antimicrobial treatments) and had been selected in preliminary experiments from an initial pool of four potential candidates for such genes (also including *16S rRNA* and *rpoB*). The efficiency (%) of each qPCR reaction (i.e., of each primer pair) had been also initially determined [49] (Table 1). Each qPCR reaction was performed in triplicate, while the data derived from a total of 1296 qPCR reactions were analyzed. These were the result of 36 different RNA/cDNA samples (i.e., 2 bacterial strains × 3 antimicrobials × 2 treatments (with and without antimicrobial exposure) × 3 biological repetitions) × 12 genes/sample × 3 technical replicates/gene.

### 2.7. Statistical Analyses for Differential Gene Expression

For each tested bacterial strain and antimicrobial, unpaired two-tailed Student’s *t*-tests were applied to the data to check for any significant difference in the expression of each target gene (expressed as log_2_(fold difference)) between the two treatments (i.e., with and without antimicrobial exposure). The same tests were also applied to check for any significant difference in the expression of each target gene between the two bacterial strains. All these tests were performed using the relevant function of Excel^®^ module of the Microsoft^®^ Office 365 suite (Redmond, WA, USA). Statistically significant expression differences were recorded at a *P* level of < 0.05. However, biologically significant ones were considered only those that in parallel presented a ǀ log_2_(fold difference) ǀ ≥ 1 between the two treatments [52].

## 3. Results and Discussion

All the antimicrobials applied here were previously verified for their strong killing efficiency against *L. monocytogenes* cells, as well as many other detrimental microorganisms [36,53,54]. Nevertheless, foodborne *L. monocytogenes* isolates displaying resistance to BAC [55,56] and enough times in parallel to other drugs, such as antibiotics and some other toxic compounds, have also been described [57,58]. Alarmingly, *L. monocytogenes* strains that are resistant to AMP have also been recovered from foods, mainly animal products probably due to the intensive use of antibiotics in animal farms [59,60,61,62]. Regarding THY and to the best of our knowledge, there are not any data available showing an increase in resistance or tolerance of *L. monocytogenes* cells following their sublethal habituation. Nevertheless, there are still some previous studies showing adaptive responses and increased survival of other bacteria following exposure to sublethal concentrations of even that natural monoterpenoid phenol [63,64]. The MIC of AMP against both bacterial strains was found equal to 0.125 μg/mL. This is a value similar to those described in the literature for that antibiotic and bacterial species [53,65]. Similarly, the MICs of BAC and THY previously determined equal to 2 and 78.1 μg/mL, respectively, against both strains [39], were similar to the ones previously reported for those compounds against that pathogenic species [56,66]. Surely, all those specific MIC values do not denote any resistance of the two strains employed here, thus confirming their initial sensitivity against all three antimicrobials. For the subsequent sublethal treatments, stationary phase cells of each serovar were exposed against a selected super-MIC (still sublethal) value of each antimicrobial. The specific concentrations tested had thus been previously shown to not cause any significant reduction in the numbers of viable and culturable cells of each strain (data not presented). Thus, all the subsequent RNA extractions were done starting from equal bacterial numbers (ca. 10^9^ CFU), to minimize the variability between the different treatments. Antimicrobial exposure was done at 37 °C, which is in the range of optimum temperatures for the planktonic growth of *L. monocytogenes* cells (i.e., 30–37 °C) just for not causing any additional thermal stress to the bacteria, while those latter had been left to enter a non-growing stationary phase before the antimicrobial challenges to imitate the bacterial physiological state in which increased resistance against various stresses is normally established [67].

**Figure 1:**
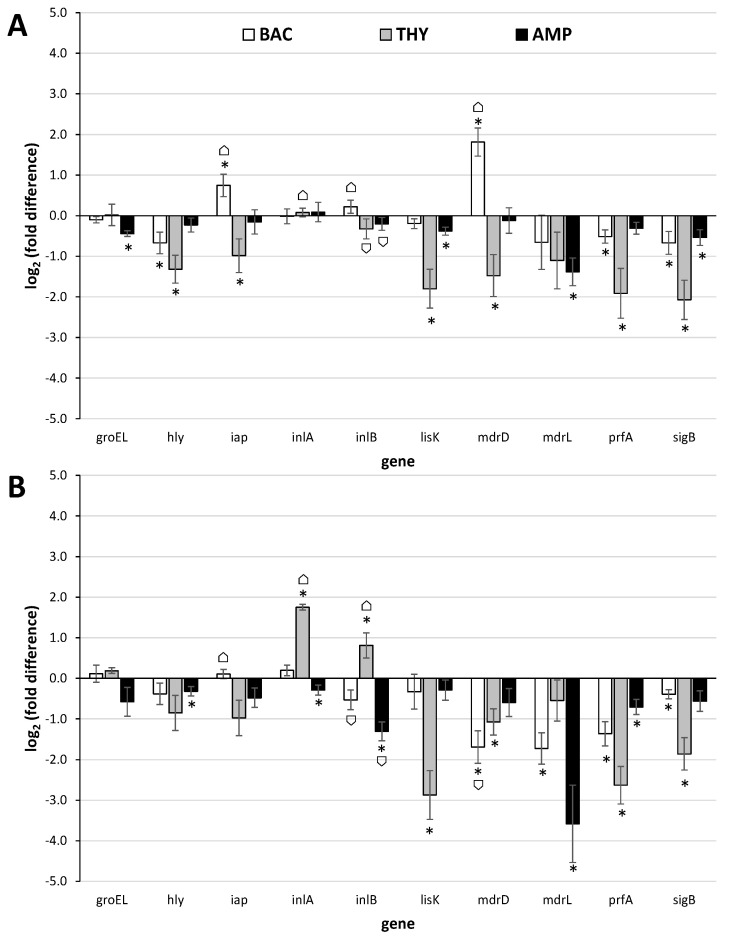
Relative quantification (log_2_(fold differences)) of the expressions of the 10 target genes (*groEL*, *hly*, *iap*, *inlA*, *inlB*, *lisK*, *mdrD*, *mdrL*, *prfA*, *sigB*) at the 2 *L. monocytogenes* strains (**A**) AAL20066 (ser. 1/2a) and (**B**) AAL20074 (ser. 4b), following their sublethal exposure (for 2 h at 37 °C) to BAC (4.0 μg/mL; □), THY (312.5 μg/mL; 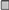) or AMP (0.5 μg/mL; 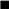), in comparison to the untreated controls (no antimicrobial exposure). Each bar represents the mean ± standard errors (*n* = 9). The statistically significant differences in genes’ expressions relative to the controls appear as asterisks (*) above the bars, while ⌂ denote the statistically significant differences in genes’ expressions between the two strains (*P* < 0.05).

The log_2_(fold differences) in genes’ expressions for both strains and all three antimicrobials are shown in Figure 1. In general, the expression of most of the studied genes remained either stable or was significantly downregulated following the antimicrobial exposure, with some strain-specific differences to be yet recorded. THY was the compound that provoked downregulation of most of the studied genes, significantly limiting the expression of 6/10 genes in one strain (ser. 1/2a), and 4/10 genes in the other strain (ser. 4b), including those coding for the master regulators of stress response and virulence (SigB and PrfA, respectively), in both strains (Figure 1 and Table S1). In agreement, sub-inhibitory THY concentration (0.50 mM) was previously described to reduce the expression of some key virulence genes in three *L. monocytogenes* strains and in parallel decrease their in vitro attachment to and invasion of human cells, motility, hemolysin production, and lecithinase activities [68]. Nevertheless, at the same time in the current study, the gene coding for the invasion surface protein internalin A (InlA), with crucial role in the onset of *L. monocytogenes* pathogenesis [69], was importantly (more than threefold) up regulated in ser. 4b strain (Figure 1B). Noteworthy, the same gene was also previously shown to be significantly overexpressed in the cells of another clinical isolate of *L. monocytogenes* belonging to the same serovar (Scott A strain) that survived exposure (for 1 h at 37 °C) to sublethal concentrations (40–100 μg/mL) of the essential oil of thyme [70].

Another gene with similar significant upregulation was that coding for the multidrug resistance transporter MdrD in ser. 1/2a strain following its exposure to BAC (Figure 1A). The expression of that gene was previously found to be significantly upregulated in *L. monocytogenes* cells during their intracellular growth in macrophages, over its level during growth in laboratory medium, thus suggesting an active role during infection [71]. In another study, the same gene was also found to be upregulated under acidic conditions (pH 5.0 vs. pH 7.3) [72]. Two other genes with statistically significant upregulation were *iap* in ser. 1/2a strain following exposure to BAC (Figure 1A), and *inlB* in ser. 4b stain following exposure to THY (Figure 1B). However, it should be noted that both recorded upregulations were slightly below the margin usually set for biologically significant differences (i.e., doubling or halving of mRNA transcripts in treated samples compared to the untreated ones; equal to a value of ǀ log_2_(fold difference) ǀ = 1).

The *iap* gene of *L. monocytogenes* encodes the invasion-associated surface protein p60, a highly antigenic protein necessary for septum separation and known to affect adherence of *L. monocytogenes* cells to, and their uptake by, mammalian cells [73]. Interestingly, this gene has been found to be activated during growth of the pathogen in a dry-cured ham model system under osmotic stress and incubation at 15 °C [24], while in another study, it was worryingly confirmed that this gene was still expressed after 6 months of incubation of the pathogen in artisanal cheese at −20 °C [74]. Long-term adaptation of *L. monocytogenes* EGD-e strain (ser. 1/2a) to either acidic (pH 5.5) or NaCl (4.5% *w/v*) stress has also been found to induce transcription of *iap* [27]. The *inlB* is the second gene of the two-genes internalin operon (the other being *inlA*), which has been known for several years to play an important role for the entry of *L. monocytogenes* into epithelial cells [75]. The simultaneous upregulation of both *inlA* and *inlB* genes that was observed here following exposure of ser. 4b strain to THY is surely a case for concern. On the other hand, the expression of both those genes remained rather constant at ser. 1/2a strain, without being changed following the antimicrobial exposures (independently of the applied antimicrobial) (Figure 1A). The expression of both *iap* and internalin genes in a strain-dependent manner was previously shown, by microarray, during growth of three *L. monocytogenes* strains, belonging to different serovars (1/2a, 4b, and 3c), in meat juices [22].

The expression of *groEL*, *hly*, *lisK*, and *mdrL* genes was here significantly downregulated following the exposure of *L. monocytogenes* bacteria to at least one of the three antimicrobials (i.e., BAC, THY, and AMP) (Figure 1 and Table S1). The *groEL* encodes a molecular chaperone that is among the most highly conserved proteins in nature, and this is known to be involved in the cellular general stress response. In bacteria, GroEL has been found to be synthesized at high levels following their exposure to abusive environmental conditions [76]. However, in this work, the expression of this gene did not significantly change following the antimicrobial exposure, except in strain AAL20066 after its exposure to AMP (although still occurring in levels much lower those typically set for biologically significant differences). The *hly* is a key virulence determinant in *L. monocytogenes* encoding the hemolysin Listeriolysin O (LLO), which has been extensively characterized for its crucial role in pathogenesis of listeriosis by promoting cell-to-cell spread and thus efficient bacterial dissemination during infection [77]. The *lisK* encodes the histidine kinase of the two-component signal transduction system LisRK that is involved in the growth of *L. monocytogenes* at low temperatures, as well as in the response of this bacterium to a number of antimicrobial agents, such as ethanol, hydrogen peroxide, nisin, and cephalosporins [78,79]. Nevertheless, none of the three antimicrobials tested in the present study was able to induce expression of this gene. Lastly, *mdrL* encodes a major facilitator superfamily (MFS) efflux pump that is involved in tolerance of *L. monocytogenes* to BAC [80]. However, in this work, this gene was surprisingly found to be significantly downregulated following the exposure of AAL20074 strain to BAC, as well as following the exposure of both strains to AMP.

In addition to the upregulation of *iap* and *mdrD* (following exposure of ser. 1/2a strain to BAC) and *inlA* and *inlB* (following exposure of ser. 4b strain to THY), no other gene was found to be significantly induced here following the antimicrobial exposure (Figure 1 and Table S1). In addition, it is worth noting that the two genes *sigB* and *prfA* coding for the master regulators of stress response and virulence, respectively [14], were both significantly downregulated in almost all cases (except *prfA* in strain AAL20066 and *sigB* in strain AAL20074 whose expression, although decreased, did not significantly change following exposure to AMP). This is rather reassuring since it implies that, in general, *L. monocytogenes* are not likely to induce either resistance or virulence following the exposure to one of the three antimicrobials tested here. Nevertheless, there are some other previously published studies that showed an alarming increase in the expression of some key stress response and/or virulence-associated genes following sublethal exposure of cells of that pathogenic species to some common antimicrobials [38,39,40].

In one such study, Kastbjerg et al. (2010) developed an agar-based assay to examine the effect of 11 disinfectants used routinely in the food industry (left to act from 15 to 180 min), representing 4 different groups of active components, on the expression of promoters of 4 virulence genes (*prfA*, *plcA*, *inlA*, and *hly*) in *L. monocytogenes* strain EGD [38]. Northern blot analysis was also performed to validate transcript levels. Disinfectants with the same active ingredients were found to have a similar effect on gene expression. Thus, peroxides and chlorine compounds reduced the expression of virulence genes, whereas QACs (five products tested) induced the expression of these genes. In another similar study, Rodrigues et al. (2011) used qPCR methodology to study the expression of *prfA* and another stress-response gene (*clpC*) in surviving *L. monocytogenes* biofilm cells following their 15-min exposure to 4 disinfectants (sodium hypochlorite at 800 μg/mL, a commercial BAC-containing product again at 800 μg/mL, hydrogen peroxide at 9%, and triclosan at 0.4%) [39]. The results showed that the expression of both genes was significantly increased in the surviving cells compared to the controls. Using the same methodology, Tamburro et al. (2015) evaluated the relative expression of *mdrL*, *ladR*, *lde*, *sigB* and *bcrABC* genes in 20 *L. monocytogenes* strains of either food or clinical origin, following sublethal 5-min exposure to 10 μg/mL of BAC, finding a significant association between increased BAC resistance and both *mdrL* and *sigB* overexpression [40].

Surely, the way the genes are transcribed in each bacterium is a rather complex procedure, influenced by its genetic make-up, the (changing) environments (both past and present), and their mazy interactions [81]. It is also known that genes’ expression may significantly vary between identically treated but different strains of the same bacterial species, or even stochastically among the cells within clonal populations [82]. Interestingly, that strain-dependent expression of stress response and virulence genes has been previously shown in *L. monocytogenes* [22,83] and was reconfirmed here for 4 out of the 10 tested genes (*iap*, *inlA*, *inlB*, and *mdrD*), also depending on the tested antimicrobial (Figure 1 and Table S1).

## 4. Conclusions

In general, the exposure of two foodborne *L. monocytogenes* strains, belonging to different listeriosis related serovars (i.e., 1/2a and 4b), to a selected sublethal concentration of each one of three common antimicrobials (i.e., BAC, THY or AMP) did not result in the transcriptomic induction of most of the key stress response and virulence-associated genes that were studied here. Nevertheless, the significant overexpression of the two genes of internalin operon (*inlA*, *inlB*) in one of the two strains (ser. 4b) following exposure to THY may be a cause for concern and should be further explored (e.g., in future in situ virulence studies employing cell cultures). In addition, the in-parallel implementation of high-throughput technologies able to globally explore and unravel the transcriptome of *L. monocytogenes* cells surviving biocidal actions of such and/or other common antimicrobials (e.g., through RNA sequencing; [84]) will increase our limited—for the time being—knowledge on the stress physiology of this important foodborne pathogenic bacterium, with hope to improve its control within the food chain and in clinical settings, ultimately protecting public health.

## Figures and Tables

**Table 1:** Sequences of the primers used for the in vitro quantitation of the mRNA transcripts of the ten target genes (*groEL, hly, iap, inlA, inlB, lisK, mdrD, mdrL, prfA, sigB*) and the two reference genes (*tuf*, *gap*). The amplicon size (bp) and amplification efficiency (%) of each primer pair, together with the regression coefficients (*R^2^*) of the linear standard curves constructed for the determination of each PCR efficiency, are also shown.

s/n	Gene	Locus Tag ^†^	Product Name	Gene Size (bp)	Primer Sequence ^‡^ (5’ → 3’)	Amplicon Size (bp)	Amplification Efficiency (%)	*R^2^*
1	*groEL*	lmo2068	molecular chaperone GroEL	1629	F: AAGTCCAGCGTTATGTGCGA	145	104.72	1.00
					R: CGTAGCTGGTGGTGGTACTG			
2	*hly*	lmo0202	listeriolysin O precursor	1590	F: TGCCAGGTAACGCGAGAAAT	135	93.96	1.00
					R: TGGTGCCCCAGATGGAGATA			
3	*iap*	lmo0582	invasion associated secreted endopeptidase	1449	F: GCCAGAGCCGTGGATGTTAT	178	113.63	0.99
					R: TTCTGGCGCACAATACGCTA			
4	*inlA*	lmo0433	internalin A	2403	F: AAATCCTGTGGCACCACCAA	137	95.78	1.00
					R: TTGTGCTGGCTGAATTCCCA			
5	*inlB*	lmo0434	internalin B	1893	F: CGCGAAGCCAAAACACCAAT	146	106.12	1.00
					R: TTGGCGCTGACATAACGAGT			
6	*lisK*	lmo1378	two-component sensor histidine kinase	1452	F: GATGTGCGTGATTACGGGGA	113	105.96	1.00
					R: CCGAGGCCATTACCACCTTT			
7	*mdrD*	lmo0872	antibiotic resistance protein	1167	F: ATCGCCGATGTTTAGCGGAA	113	108.26	0.98
					R: CATTCGCAAAATGCCCACCA			
8	*mdrL*	lmo2377	multidrug transporter	1212	F: CCGTTGCTTGCGCTTTATGT	117	94.03	0.97
					R: TCCCCATTTTCGCGTCATCA			
9	*prfA*	lmo0200	listeriolysin positive regulatory protein	714	F: CTGAGCTATGTGCGATGCCA	138	101.96	0.98
					R: AGCTTGGCTCTATTTGCGGT			
10	*σ^B^ (sigB)*	lmo0895	RNA polymerase sigma factor SigB	780	F: CTTCAAAGCTCGCCGCAAAT	182	105.40	1.00
					R: CCATCATCCGTACCACCAACA			
11	*tuf*	lmo2653	elongation factor Tu	1188	F: CCAATGTTGTCGCCAGCTTC	149	101.00	1.00
					R: GCAACTGGACGTGTTGAACG			
12	*gap*	lmo2459	glyceraldehyde-3-phosphate dehydrogenase	1011	F: AGCTGCTTCCATAGCTGCATT	114	95.88	0.96
					R: TTAGACGGAGCTGCTCAACG			

^†^ Corresponds to the NCBI Reference Sequence: NC_003210.1 of the complete genome of *L. monocytogenes* strain EGD-e [50]. ^‡^ Target-specific conserved primers were designed using Primer-BLAST software developed at National Center for Biotechnology Information (NCBI, Bethesda MD, 20894 USA; http://www.ncbi.nlm.nih.gov/tools/primer-blast; (Accessed on 29 August 2021) [51]).

## Data Availability

The data presented in this study are available on request from the corresponding author.

## Supplementary Materials

The following are available online at https://www.mdpi.com/article/10.3390/foods10102382/s1, Table S1: Statistically significant changes (**↑**: up regulations; ↓: down regulations; *P* < 0.05) in the expressions of the 10 target genes (*groEL, hly, iap, inlA, inlB, lisK, mdrD, mdrL, prfA, sigB*) at the 2 *L. monocytogenes* strains AAL20066 (ser. 1/2a) and AAL20074 (ser. 4b), following their sublethal exposure (for 2 h at 37 °C) to BAC (4.0 μg/mL), THY (312.5 μg/mL) or AMP (0.5 μg/mL), in comparison to the untreated controls (no antimicrobial exposure). ∞: no significant change.
