# Supplementary material for: Investigating Transcriptomic Induction of Resistance and/or Virulence in Listeria monocytogenes Cells Surviving Sublethal Antimicrobial Exposure
Source: Foods. 2021 Oct 8;10(10):2382. doi: 10.3390/foods10102382 (PMC8535302; doi:10.3390/foods10102382)
Supplement: Supplementary file 1 [file foods-10-02382-s001.zip › foods-1379036-supplementary.pdf]

Table S1. Statistically significant changes (↑: up regulations; ↓: down regulations; $P < 0.05$) in the expressions of the ten target genes (*groEL, hly, iap, inlA, inlB, lisK, mdrD, mdrL, prfA, sigB*) at the two *L. monocytogenes* strains AAL20066 (ser. 1/2a) and AAL20074 (ser. 4b), following their sublethal exposure (for 2 hours at 37 °C) to BAC (4.0 μg/mL), THY (312.5 μg/mL) or AMP (0.5 μg/mL), in comparison to the untreated controls (no antimicrobial exposure). ∞: no significant change.

s/n	Gene	BAC		THY		AMP		∞	↑	↓
		AAL20066	AAL20074	AAL20066	AAL20074	AAL20066	AAL20074			
1	*groEL*	∞	∞	∞	∞	↓	∞	5	0	1
2	*hly*	↓	∞	↓	∞	∞	↓	3	0	3
3	*iap*	↑	∞	↓	∞	∞	∞	4	1	1
4	*inlA*	∞	∞	∞	↑	∞	↓	4	1	1
5	*inlB*	∞	∞	∞	↑	∞	↓	4	1	1
6	*lisK*	∞	∞	↓	↓	↓	∞	3	0	3
7	*mdrD*	↑	↓	↓	↓	∞	∞	2	1	3
8	*mdrL*	∞	↓	∞	∞	↓	↓	3	0	3
9	*prfA*	↓	↓	↓	↓	∞	↓	1	0	5
10	σ^B (*sigB*)	↓	↓	↓	↓	↓	∞	1	0	5
	∞	5	6	4	4	6	5			
	↑	2	0	0	2	0	0			
	↓	3	4	6	4	4	5			

Differential behavior in genes' expressions between the two strains.

Statistically significant differences in genes' expressions between the two strains ($P < 0.05$).
